# Comparative effects of vitamin D and vitamin C supplementations with and without endurance physical activity on metabolic syndrome patients: a randomized controlled trial

**DOI:** 10.1186/s13098-018-0384-8

**Published:** 2018-11-08

**Authors:** Halgord Ali M. Farag, Mohammad Javad Hosseinzadeh-Attar, Belal A. Muhammad, Ahmad Esmaillzadeh, Abdel Hamid El Bilbeisi

**Affiliations:** 1 Department of Clinical Nutrition, School of Nutritional Sciences and Dietetics, Tehran University of Medical Science, International Campus (TUMS-IC), Tehran, Iran; 2 Halabja Technical Institute, Sulaimani Polytechnic University, Kurdistan, Iraq; 3 Department of Community Nutrition, School of Nutritional Sciences and Dietetics, Tehran University of Medical Sciences, Tehran, Iran

**Keywords:** Endurance physical activity, IDF, Metabolic syndrome, Vitamin C, Vitamin D

## Abstract

**Objective:**

Vitamin D and C levels have inverse relation with the metabolic syndrome components and they are used as antioxidant supplements during enduring metabolic activities. In the present study, we hypothesized that the intake of vitamin D and/or C with endurance physical activity might reduce the risk of metabolic syndrome.

**Methods:**

A randomized control study recruited 180 participants of both genders, aged between 30 and 50 years. The participants were assigned into six groups receiving different doses of vitamin D or vitamin C with or without physical activities. Data were collected over a period of 3 months, and the results were analyzed using SPSS version 20.

**Results:**

Variations in the effect of the supplements on various body variables including: Fasting plasma glucose, total cholesterol, low-density lipoprotein cholesterol and blood pressure, showed that vitamin D has more influence compared to vitamin C. However, vitamin D and C supplements do not have any effect on weight when consumers are undergoing endurance physical exercise. But vitamin C consumer group has more effect in waist circumference, triglyceride, and high-density lipoprotein, as compared to vitamin D consumer group.

**Conclusion:**

We conclude that, consumption of vitamin D or vitamin C supplements may improves the life of metabolic syndrome patients. However, the combination of physical activities and vitamin supplements maximize the effect, and this combination should be recommended.

*Trial registration* WHO-ICTRP IRCT20161110030823N2. Registered 01 February 2018. http://apps.who.int/trialsearch/Trial2.aspx?TrialID=IRCT20161110030823N2

## Background

Metabolic syndrome (MetS) is a constellation of abnormal cardio metabolic factors that increase risk of cardiovascular disease (CVD) and type 2 diabetes mellitus [1]. MetS is a major health problem worldwide; based on the International Diabetes Federation (IDF) appreciation about one quarter of the world’s adult population have MetS [1]. MetS is among dangerous syndromes, which increases the danger of being overtaken by various diseases including CVD, diabetes, dyslipidemia, stroke, osteoarthritis, some type of cancers and mortality [2]. MetS imposes heavy expenses to sanitary therapeutic system and it generally reduces life quality [3]. In fact, insulin resistance and central obesity are considered the main causes of MetS [1–3]. In addition, its appear that demographic, lifestyle, and social factors are affecting MetS [2]. Indeed, it has been reported that cigarette consumption and high body mass view are among independent and amendable risk factors for MetS [2]. Nowadays, recognition of considerable challenges related to obesity and its therapeutic solutions all over the lifetime have resulted in great efforts spent toward obesity inhibition [4]. Furthermore, doing regular athletic activities and consuming the antioxidants are among the advised solutions, which are not only affecting the total safety of body, but also affect brain performance [2]. Some of previous studies have reported that people who experience delayed performance physically showed improvement with supervised physical fitness exercises, and the health of people suffering from metabolic diseases improved with an increase in antioxidant intake into their system [4, 5]. Antioxidant supplementations especially vitamin C relieve the body off the stress associated with MetS and vitamin D increase the antioxidant capacity [4, 5]. Some of previous studies show a significant relationship between the amount of vitamin D intake and muscle fatigue; supplements are likely to improve the muscle fatigue through the biological roles that these nutrients play [6, 7]. A change in vitamin D from the normal range alters the muscle performance and activity significantly, especially when exercises of different intensities are involved [8–11]. Elsewhere studies have reported a reduction of death with frequent intake of vitamin D as well as engaging in aerobic exercises [12–16]. Regarding the relation between serum level of vitamin D and MetS components, different studies have been accomplished in some of which this relation has been confirmed [17–19]. It has been shown that low level of serum vitamin D has inverse relation with weight gain, body mass index (BMI), but it does not have significant relation with other components of MetS [17]. Accumulating literature also link vitamin D studies with bones while comparing BMI and general weight gains [14–16]. On the other hand, vitamin C however, has completely different functions in an in vitro experiments and in humans [20–22]; some previous studies show that, the metabolism of glucose in the insulin resistant people was improved, and the blood pressure was lowered in patients who took vitamin C supplementation [23]. Furthermore, vitamin C and vitamin E supplementation are shown to prevent molecular regulators that trigger the sensitivity of insulin as well antioxidant defense mechanisms through physical activities [24]. Moreover, endurance exercises produce reactive nitrogen and oxygen through the mitochondria [25]. Physical exercises on a regular basis promote the health of the individuals and its play a vital role in treating MetS patients [26–28]. In conclusion, the prevalence of MetS is raising worldwide [1]. In addition, vitamin D and vitamin C levels have inverse relation with some of the MetS components such as (BMI, insulin resistant, and high blood pressure) [17, 23], and they are used as antioxidant supplements during enduring metabolic activities [4, 5]. Therefore, understanding the association between the effects of vitamin D and vitamin C supplementations with and without endurance physical activity (PA) on various components of MetS may be helpful in reducing MetS-related premature mortality and improve life quality among MetS patients. Our study was conducted to examine the effects of vitamin D and vitamin C supplementations with and without endurance PA on various components of MetS among MetS patients.

## Methods

### Research design and study population

**Fig. 1:**
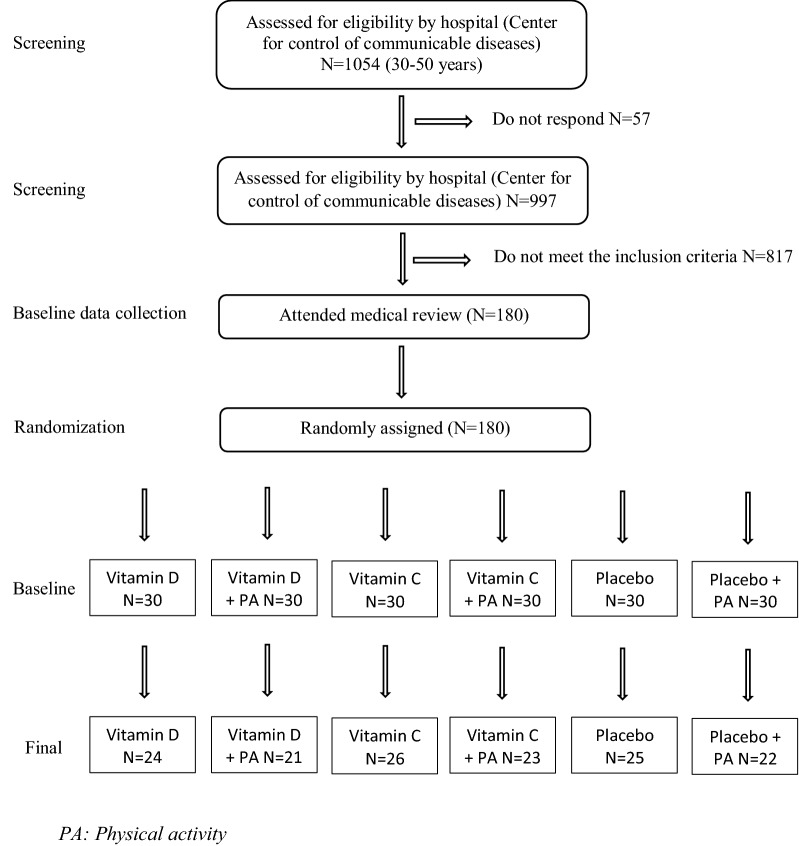
Flow chart for inclusion and exclusion in the study

The study design for this research is a randomized controlled trial (RCT) [29]. Based on the suggested formulas of this model, the study participants were recruited on the foundation of developed inclusion/exclusion criteria [30]. The subjects underwent a 12 weeks’ treatment program (01 March 2016 to 23 May 2016). Participants were randomly assigned into six Groups (Fig. 1): (1) Vitamin C group: who took only 500 mg/day vitamin C supplements [Morning Time]; (2) Vitamin C plus PA group either morning 7:30 A.M. or afternoon after 3:00 P.M.: who participated in 30 min/day of endurance PA and also took 500 mg/day vitamin C supplements. (3) Vitamin D group: who took only 2000 IU/day vitamin D supplements (Morning Time); (4) Vitamin D plus PA group either morning 7:30 P.M. or afternoon after 3:00 P.M.: those who participated in 30 min/day of endurance PA and also took 2000 IU/day vitamin D supplements. (5) Placebo group, who participated in 30 min/day of endurance PA and took a placebo, (6) final group; did not participate in 30 min/day of endurance PA, but took a placebo. Both vitamins and placebo were obtained from Osweh manufacture Iran-Tehran and was prepared to feature the same shape, odor and size of the supplements.

### Sample size and sample determination

In the present study, the sample size was calculated using a previously described formula for parallel clinical trials n = 2 [(z1 − α/2 + z1 − β) 2. s2]/d2 [31]. In this formula, n is number of participants in each group. For estimating sample size, we considered type one (α) and type two errors (β) of .05 and .20 (Power = 80%) respectively, and fasting plasma glucose levels as a key variable. Based on a previous study [31], standard deviation (SD) of plasma glucose levels was 8 mg/dL and the difference in mean (d) was considered to be 5 mg/dL. Where α = .95, β = 20%, study power = 80%, d = 5, and SD = 8.

$${\text{n}} = \frac{{2 \left( {1.96 + 0.85} \right)^{2} \left( 8 \right)^{2} }}{{\left( 7 \right)^{2} }} = 21$$

We reached the sample size of 21 subjects for each group. In addition, to consider probable dropouts, 30 patients were included in each group. At the end, a total of 180 patients with MetS were included in the present study. Participants were distributed into six groups as shown in Fig. 1.

### Inclusion and exclusion criteria

Eligibility criteria for participants having MetS, according to IDF definition [1] and the age between 30 and 50 years, both males and females. Individuals who took supplements containing vitamins D and C in the last 3 years were not included in this study. Individuals with type I and type II diabetes who were taking oral hypoglycemic agents or injecting insulin, or any medical therapy affecting the result, smokers, individuals with heart failure, and those who are suffering from renal problems, also individuals with malabsorption syndrome, pregnancy and lactating mothers were all excluded from the study. The exclusion list included patients with history of bariatric surgery and those who are currently using weight-loss medications as illustrated in (Fig. 1).

### Data collection

Data collection was performed in the community health and control of communicable disease center. At baseline, standardized general questionnaires were completed for each subject [32]. Additional information regarding demographic, and medical history variables was obtained with an interview-based questionnaire. Past history and any previous treatment for certain disease including hypertension, diabetes, high cholesterol, supplement used, family history of obesity, family history of diabetes, family history of hypertension as well as PA patterns was also recorded.

### Anthropometric measurements

Weight (kg) was measured while the subjects with minimal clothe without shoes using a digital scales and records to the nearest 100 g. Height (m) was measured in a standing position, without shoes, using a tape measure. BMI was calculated as weight in kilograms divided by height in meters squared [33]. Waist circumference (WC) was measured at the mid-way between the lower border of the ribs and the iliac crest with the subject in standing position. During the measurement process, it was critical to use the same technician for the purpose of error reduction.

### Biochemical analysis

The participants were asked to produce a blood sample of 10 cc, which was collected from the participant at base line 0 and 12 weeks of the study. The blood was collected after overnight fasting for approximately 12 h. The blood samples were taken using the protocol outlined in [34]. Variables of concern in this test were the fasting plasma glucose (FPG) that were measured on the day of blood collection as outlined in [35]. The blood sample was investigated for FPG mg/dL, total cholesterol (TC) mg/dL, triglyceride (TG), low-density lipoprotein cholesterol (LDL-C) mg/dL, high-density lipoprotein cholesterol (HDL-C) mg/dL, vitamin D (ng/mL) and vitamin C (ng/mL).

### Assessment of blood pressure

The systolic and diastolic blood pressure was taken from the left arm (mmHg) in the morning during each interview (From baseline and end of study) using the calibrated mercury sphygmomanometer [36]. Participants were seated after relaxing for at least fifteen minutes in a quiet environment, empty bladder. The average of the measurements was recorded.

### Assessment of PA

Two times a week for climbing (around 2 h each time) and two times a week for running (around 1 h in the afternoon between 3 and 5 P.M.). Approximately 6 h per week all together [37].

### Statistical analysis

We used Kolmogorov–Smirnov test to examine the normal distribution of variables. The analyses were done based on intention-to-treat approach. Baseline general characteristics among different groups were examined using one-way ANOVA for continuous variable and a Chi square test for categorical variables. To determine the effects of vitamin D and vitamin C supplementation and endurance PA on metabolic criteria, we used one-way ANOVA. We used Tukey’s post hoc comparisons to identify pairwise differences when we reached a significant finding in ANOVA. P < .05 was considered as statistically significant. All statistical analyses were done using SPSS version 20.

## Results

In the present study, 180 patients with MetS were recruited: vitamin D (n = 30), “vitamin D plus 30 min/day PA” (n = 30), vitamin C (n = 30), “vitamin C plus 30 min/day PA” (n = 30), placebo (n = 30) and “placebo plus 30 min/day PA” (n = 30) groups. The study procedure and the flow chart for inclusion and exclusion in the study is shown in Fig. 1. In did, 39 participants excluded from the study because of the following reasons: Five became pregnant, nine did not follow recommended PA, seven had poor compliance to vitamin D supplements, 10 had poor vitamin C supplement, and 8 did not complete the trial. Finally, 141 subjects remained in the study. We included the data for all 141 participants in the baseline and final analysis. The characteristics of the study participants are presented in Table 1. The distribution of participants in terms of mean age among different study groups was not significant (P value > .05). The percentage of gender allocation, family history of obesity, family history of diabetes mellitus and family history of hypertension was not significantly different across the intervention groups (P value > .05 for all). These data suggest that the demographic information at baseline studies is evenly distributed. In addition, the results of the present study demonstrated that, baseline serum vitamin D and vitamin C levels showed no significant difference among groups (P value > .05). There were also no significant differences among the groups in terms of weight, BMI, WC, FPG, SBP, DBP and TC (P value > .05 for all). However, participants who received vitamin C supplements had higher serum levels of TG compared with those who received vitamin D plus PA, vitamin D plus PA and placebo or “placebo plus PA” (P < .001). Participants in the “vitamin D plus PA and placebo plus PA” groups had higher values of HDL-C compared with those in the other groups (P = .046). Furthermore, participants in the vitamin C group had lower levels of LDL-C compared with those in the “placebo plus PA” group (P = .007). Moreover, end of trial means of metabolic characteristic measures among study groups are shown in Table 2. We observed a significant increase in mean serum vitamin D concentrations, in participants who received vitamin D, (Vitamin D group from 10.8 ± 2.8 to 23.2 ± 4.9 ng/mL, or “vitamin D plus PA” from 10.4 ± 3.2 to 29 ± 5.5 ng/mL, P < .001 for all) and a significant increase in vitamin C supplementation also observed (Vitamin C group from .9 ± .4 to 1.4 ± .3 ng/mL or “vitamin C plus PA” from .8 ± .3 to 1.7 ± .3 ng/mL, P < .001 for all). There was also a significant increase in serum levels of vitamin D in those who received “placebo plus PA” (11 ± 4 vs. 18.9 ± 4.5 ng/mL at the end of the study, P < .001). No significant changes in serum levels of vitamin D and vitamin C were seen in participants in the placebo groups (P value > .05). End of trial supplementation did not significantly affect means of anthropometric measures and blood pressure among all groups (P value > .05). Whereas, One-way ANOVA of the end of study FPG showed significant difference between groups (P value < .05), and biochemical indicators across all study groups after intervention shows that, subjects in vitamin D group had lower serum levels of TC compared with the other groups (P < .001) and highest level of TC was observed in placebo group compared to other groups. In terms of TG, vitamin C intake resulted in higher serum levels of TG compared with the other groups (P < .001). Additionally, end of trial means of serum levels of LDL-C was significantly lower in the vitamin D group compared with all of other groups (P < .001). And the participants in vitamin C plus PA group had higher serum levels of HDL-C compared with other groups (P < .001). On the other hand, changes in metabolic criteria across study groups are presented in Table 3. There was a marginally significant difference in changes of BMI, WC, TC, TG, LDL-C, and HDL-C among all groups (P value < .05 for all). No significant changes in serum levels of FPG, SBP and DBP were seen among all groups (P value > .05 for all). Finally, multiple comparison of metabolic criteria across the study groups are presented in Table 4. According to multiple comparison using Tukey method across the study groups, the mean difference change of BMI only lowered in those who took vitamin D and did exercise compared with those who took vitamin D alone (− 1.4 ± .4, P = .029). However, the change was not significant compared to placebo. In addition, WC was significantly lowered in the vitamin C plus PA group compared to placebo (− 2.8 ± 1.0, P = .041) and vitamin D alone (− 4.2 ± 1.0, P < .001). Our results demonstrate that, taking vitamin C with exercise lowered WC to higher level comparing to those who took vitamin C alone (2.9 ± 1.0, P = .021). Taking placebo plus PA also lowered WC compared to vitamin D supplement alone (3.5 ± 1.0, P = .006). Besides, taking vitamin C and doing exercise remarkably lowered WC comparing to those who took vitamin D and exercised (3 ± 1.0, P = .041). Regarding change in lipid profiles a significant changes in serum levels of TC were seen following vitamin D or “vitamin D plus PA” than that in the placebo group (24 ± 8, P = .037; 24 ± 8.2, P = .050) respectively. Beside, either taking vitamin C and “vitamin C plus PA” both significantly lowered TG in comparison to placebo (33.5 ± 10.1, P = .027; 36.2 ± 11.1 P = .017). Interestingly, Table 4 showed that mean of HDL-C was higher in those who took vitamin C and doing PA compared to those vitamin D plus PA (16.2 ± 4.0, P = .002) and placebo plus PA (16.3 ± 4.0, P = .001). Vitamin C with exercise lowered HDL-C more in than taking vitamin C alone (14.2 ± 3.9, P = .005), Vitamin D alone (15.6 ± 3.9, P = .002) and vitamin D plus PA (16.2 ± 4.0, P = .002). Even so, only vitamin D supplement lowered LDL-C more in compared with vitamin C supplement groups (23.4 ± 8.2, P = .055).

**Table 1:** Baseline characteristics of the study participants

Variables	Groups (n = 141)						P value^g^
	Vit D (n = 24)^a^	Vit D + PA (n = 21)^b^	Vit C (n = 26)^c^	Vit C + PA (n = 23)^d^	Placebo (n = 25)^e^	Placebo + PA (n = 22)^f^	
Age (years)	40.5 ± 5.9	40.4 ± 5.9	41.2 ± 5.8	40.8 ± 5.8	42.6 ± 5.6	41.6 ± 6.4	.812
Female (%)	67	67	62	70	48	59	.725
Family history of obesity (%)	75	81	65	65	76	50	.314
Family history of DM (%)	50	67	46	35	60	41	.321
Family history of BP (%)	58	62	46	57	60	41	.753
Weight (kg)	84.2 ± 16.7	81.7 ± 9.6	81 ± 13.2	79.7 ± 13.6	82.3 ± 14	74.9 ± 12.7	.354
BMI (kg/m^2^)	33.1 ± 5.9	33.4 ± 4.3	32 ± 6.1	32.3 ± 5.9	32.8 ± 4.3	30.1 ± 4.7	.475
WC (cm)	109.2 ± 8	108.5 ± 7.7	108.2 ± 10.4	107.8 ± 8.7	107.6 ± 9.6	111.5 ± 10.2	.824
FPG (mg/dL)	108 ± 17.1	106 ± 11.8	106.6 ± 19.9	104 ± 13.7	110.6 ± 17.2	106.2 ± 18.1	.871
SBP (mmHg)	127.1 ± 11.5	129 ± 12.7	130.2 ± 12	129 ± 11.4	125.6 ± 14.5	128.6 ± 10.8	.810
DBP (mmHg)	79.8 ± 9.5	83.6 ± 9.4	81.3 ± 8.3	79.3 ± 7.1	80 ± 6.9	82.5 ± 5.9	.421
TC (mg/dL)	173.5 ± 60.8	194.7 ± 32.2	174.8 ± 41.5	178.3 ± 29.8	185.9 ± 39	149 ± 35.8	.326
TG (mg/dL)	229.3 ± 113.8	184.5 ± 98.5	268.4 ± 107.2^*^	176.1 ± 87.4	147.4 ± 43	161.7 ± 70.1	.001
LDL-C (mg/dL)	120.7 ± 64.4^†^	149.6 ± 35.8	114.5 ± 47.5	135.5 ± 32.7	150.4 ± 39.8	153.5 ± 38.4	.007
HDL-C (mg/dL)	34.9 ± 17.3	40.9 ± 14.4	33 ± 13.5	37.6 ± 11^**^	30 ± 8.5	40.8 ± 16.5	.046
Vit D (ng/mL)	10.8 ± 2.8	10.4 ± 3.2	–	–	12.2 ± 4	11 ± 4	.434
Vit C (ng/mL)	–	–	.9 ± .4	.8 ± .3	1 ± .3	.9 ± .3	.320

Data are mean ± standard deviation (SD)

Vit D, vitamin D; PA, physical activity; Vit C, vitamin C; DM, diabetes mellitus; BP, blood pressure; BMI, body mass index; WC, waist circumferences; DM, diabetes mellitus

^*^P < .05 compared with the placebo group, using Tukey’s test

^**^P < .05 compared with the vitamin D and “placebo plus physical activity” groups, using Tukey’s test

^†^P < .05 compared with the other groups, using Tukey’s test

^a^Receiving 2000 IU vitamin D per day

^b^Receiving 2000 IU vitamin D per day plus 30 min endurance physical activity

^c^Receiving 500 mg vitamin C per day

^d^Receiving 500 mg vitamin C per day plus 30 min endurance physical activity

^e^Receiving one placebo per day

^f^Receiving one placebo per day plus 30 min endurance physical activity

^g^Obtained from ANOVA or Chi square test, where appropriate

**Table 2:** End of trial means of metabolic characteristic across the study groups

Variables	Groups (n = 141)						P value^g^
	Vit D (n = 24)^a^	Vita D + PA (n = 21)^b^	Vit C (n = 26)^c^	Vit C + PA (n = 23)^d^	Placebo (n = 25)^e^	Placebo + PA (n = 22)^f^	
Weight (kg)	84.9 ± 16.7	79 ± 11	79.5 ± 12.9	77.9 ± 13.4	82.3 ± 13.8	73.6 ± 12.5	.124
BMI (kg/m^2^)	33.3 ± 6	32.2 ± 4	311.4 ± 6	31.7 ± 5.6	32.8 ± 4.2	29.7 ± 4.7	.231
WC (cm)	109.1 ± 8.2	107.2 ± 7.5	107 ± 9.9	103.6 ± 8.4	106.2 ± 9.7	107.9 ± 10.4	.442
FPG (mg/dL)	97.8 ± 7.7	97.7 ± 8.7	105 ± 13.3	100.7 ± 8.4	108 ± 19.2	98.9 ± 10.7	.012
SBP (mmHg)	121.3 ± 9.2	126.2 ± 8.9	129.2 ± 8.6	173 ± 252.4	126.6 ± 12	123 ± 12	.514
DBP (mmHg)	80.1 ± 5.8	81.7 ± 5.3	81.9 ± 5	79.6 ± 4.5	81.6 ± 6.7	80.2 ± 6.7	.643
TC (mg/dL)	160.5 ± 33.4	81.7 ± 31.3	180.2 ± 31.9	175.1 ± 29.9	196.8 ± 39.4	184.8 ± 28.1	.009
TG (mg/dL)	233.8 ± 97	178 ± 80.8	246.1 ± 90.3	151.2 ± 53.8	158.6 ± 35.4	155.6 ± 57.7	.001
LDL-C (mg/dL)	107 ± 36.6	138.3 ± 31.4	124.3 ± 39.6	134.5 ± 28.2	158.8 ± 39	145.8 ± 30.2	.001
HDL-C (mg/dL)	33.7 ± 10.6	39.1 ± 10	33.2 ± 10.1	51.9 ± 20.6	31.8 ± 7.1	38.9 ± 9.8	.001
Vit D (ng/mL)	23.2 ± 4.9	29 ± 5.5	–	–	12.6 ± 4	18.9 ± 4.5	.001
Vit C (ng/mL)	–	–	1.4 ± .3	1.7 ± .3	.9 ± .4	1.1 ± .3	.001

Data are mean ± standard deviation (SD)

PA, physical activity; BMI, body mass index; WC, waist circumferences; FPG, fasting blood sugar; SBP, systolic blood pressure; DBP, diastolic blood pressure; TC, total cholesterol; TG, triglycerides; HDL-C, high density lipoprotein cholesterol; LDL-C, low-density lipoprotein cholesterol

^a^Receiving 2000 IU vitamin D per day

^b^Receiving 2000 IU vitamin D per day plus 30 min endurance physical activity

^c^Receiving 500 mg vitamin C per day

^d^Receiving 500 mg vitamin C per day plus 30 min endurance physical activity

^e^Receiving one placebo per day

^f^Receiving one placebo per day plus 30 min endurance physical activity

^g^Obtained from ANOVA or Chi square test, where appropriate

**Table 3:** Change in changes in metabolic criteria across a cross the study groups

Variables	Groups (n = 141)						P value^g^
	Vit D (n = 24)^a^	Vita D + PA (n = 21)^b^	Vit C (n = 26)^c^	Vit C + PA (n = 23)^d^	Placebo (n = 25)^e^	Placebo + PA (n = 22)^f^	
Weight (kg)	.4 ± 1.3	− 2.8 ± 7.9	− 1.6 ± 2.2	− 1.8 ± 4.3	− .03 ± 1.3	− 1.2 ± 1	.058
BMI (kg/m^2^)	.16 ± .5	− 1.3 ± 3.5	− .6 ± .8	− .6 ± 1.3	− .01 ± .5	− .5 ± .4	.031
WC (cm)	− .04 ± 2.1	− 1.2 ± 2.2	− 1.2 ± 2.3	− 4.2 ± 6.1	− 1.4 ± 3.6	− 3.6 ± 1.4	.001
FPG (mg/dL)	− 10.2 ± 15.9	− 8.3 ± 16.2	− 1.7 ± 11	− 3.3 ± 14.3	− 2.6 ± 19.7	− 7.4 ± 14.6	.325
SBP (mmHg)	− 5.8 ± 7.1	− 2.9 ± 9.7	− .1 ± 8	44.1 ± 251.1	1 ± 8.2	− 5.7 ± 7.9	.552
DBP (mmHg)	1 ± 6.9	− 1.9 ± 7.7	.6 ± 7.4	.2 ± 8.2	1.6 ± 8.7	− 2.3 ± 6.3	.434
TC (mg/dL)	− 13 ± 42.3	− 13 ± 16.7	5.4 ± 24.9	− 3.1 ± 22.4	10.9 ± 32.8	− 9.3 ± 17	.012
TG (mg/dL)	4.6 ± 32.9	− 6.4 ± 29.4	− 22.3 ± 31.2	− 25 ± 68.8	11.2 ± 29.8	− 6.1 ± 18.8	.006
LDL-C (mg/dL)	− 13.7 ± 43.8	− 11.3 ± 19.8	9.8 ± 24.8	− 1 ± 27.8	8.3 ± 30.9	− 7.7 ± 17.9	.017
HDL-C (mg/dL)	− 1.3 ± 12.9	− 1.9 ± 13.4	.1 ± 11.1	14.3 ± 20	1.7 ± 7.2	− 1.9 ± 13.9	.001
Vit D (ng/mL)	12.4 ± 4.3	18.6 ± 6.8	–	–	.5 ± 1.3	7.9 ± 2.8	.001
Vit C (ng/mL)	–	–	.5 ± .5	.9 ± .4	− .07 ± .5	.1 ± .5	.001

Data are mean ± standard deviation (SD)

PA, physical activity; BMI, body mass index; WC, waist circumferences; FPG, fasting blood sugar; SBP, systolic blood pressure; DBP, diastolic blood pressure; TC, total cholesterol; TG, triglycerides; HDL-C, high-density lipoprotein cholesterol; LDL-C, low-density lipoprotein cholesterol

^a^Receiving 2000 IU vitamin D per day

^b^Receiving 2000 IU vitamin D per day plus 30 min endurance physical activity

^c^Receiving 500 mg vitamin C per day

^d^Receiving 500 mg vitamin C per day plus 30 min endurance physical activity

^e^Receiving one placebo per day

^f^Receiving one placebo per day plus 30 min endurance physical activity

^g^Obtained from ANOVA or Chi square test, where appropriate

**Table 4:** Multiple comparison of BMI, WC, TC, TG, LDL-C and HDL-C across the study groups

Multiple comparisons: Tukey HSD											
Dependent variables		Mean difference (I–J)	Std. error	Sig.	95% confidence interval		Mean difference (I–J)	Std. error	Sig.	95% confidence interval	
(I) Group	(J) Group				Lower bound	Upper bound				Lower bound	Upper bound
Changes in BMI							Change in WC				
Vit D^a^	Vit D + PA	1.4^*^	.45	.029	.09	2.72	1.19	.99	.831	− 1.6	4.07
	Vit C	.74	.43	.512	− .50	1.99	1.18	.94	.802	− 1.5	3.91
	Vit C + PA	.79	.44	.473	− .48	2.08	4.17^*^	.97	.001	1.3	6.98
	Placebo	.16	.43	.991	− 1.09	1.42	1.35	.95	.714	− 1.3	4.11
	Placebo + PA^f^	.63	.44	.722	− .66	1.93	3.54^*^	.98	.006	.70	6.39
Vit C^c^	Vit D + PA	.66	.44	.675	− .63	1.95	.007	.97	1.01	− 2.8	2.83
	Vit C + PA	.05	.43	1.04	− 1.20	1.31	2.98^*^	.95	.021	.22	5.74
	Placebo	− .57	.42	.755	− 1.81	.65	.16	.93	1.03	− 2.52	2.86
	Placebo + PA	− .11	.44	1.01	− 1.38	1.16	2.36	.96	.142	− .43	5.15
Vit D + PA^b^	Vit C + PA	− .60	.45	.772	− 1.93	.72	2.97^*^	1.00	.041	.07	5.88
	Placebo	− 1.23	.45	.074	− 2.54	.06	.16	.98	1.01	− 2.68	3.01
	Placebo + PA	− .77	.46	.551	− 2.11	.56	2.35	1.01	.192	− .58	5.29
Vit C + PA^d^	Placebo	− .63	.43	.705	− 1.90	.64	− 2.81^*^	.96	.041	− 5.60	− .034
	Placebo + PA	− .16	.45	.998	− 1.47	1.14	− .62	.99	.987	− 3.49	2.24
Placebo^e^	Placebo + PA	.46	.44	.901	− .82	1.75	2.19	.97	.221	− .62	5.00
Change in TC							Change in TG				
Vit D^a^	Vit D + PA	− .047	8.33	1.00	− 24.15	24.05	10.96	11.48	.931	− 22.23	44.16
	Vit C	− 18.38	7.89	.190	− 41.21	4.44	26.86	10.87	.141	− 4.59	58.31
	Vit C + PA	− 9.86	8.14	.830	− 33.40	13.66	29.52	11.21	.096	− 2.89	61.95
	Placebo	− 23.92^*^	7.97	.037	− 46.97	− .86	− 6.64	10.98	.990	− 38.40	25.10
	Placebo + PA^f^	− 3.72	8.23	.998	− 27.53	20.08	10.67	11.34	.935	− 22.12	43.47
Vit C^c^	Vit D + PA	18.33	8.18	.227	− 5.33	42.00	− 15.89	11.27	.721	− 48.49	16.70
	Vit C + PA	8.51	7.98	.894	− 14.57	31.60	2.66	11.00	1.00	− 29.14	34.47
	Placebo	− 5.53	7.81	.981	− 28.13	17.059	− 33.51^*^	10.76	.027	− 64.63	− 2.38
	Placebo + PA	14.65	8.08	.461	− 8.71	38.02	− 16.18	11.13	.694	− 48.37	16.00
Vit D + PA^b^	Vit C + PA	− 9.82	8.42	.852	− 34.16	14.52	18.56	11.60	.600	− 14.97	52.09
	Placebo	− 23.8^*^	8.25	.050	− 47.74	.005	− 17.61	11.37	.634	− 50.50	15.27
	Placebo + PA	− 3.67	8.51	.998	− 28.28	20.92	− .29	11.72	1.00	− 34.19	33.60
Vit C + PA^d^	Placebo	− 14.05	8.06	.506	− 37.35	9.25	− 36.17^*^	11.10	.017	− 68.28	− 4.07
	Placebo + PA	6.14	8.32	.977	− 17.91	30.19	− 18.85	11.46	.571	− 51.98	14.28
Placebo^e^	Placebo + PA	20.19	8.15	.139	− 3.38	43.77	17.32	11.23	.638	− 15.15	49.80
Change in LDL-C							Change in HDL-C				
Vit D^a^	Vit D + PA	− 2.36	8.68	1.00	− 27.46	22.74	.60	4.03	1.00	− 11.07	12.27
	Vit C	− 23.47*	8.22	.055	− 47.25	.30	− 1.39	3.82	.992	− 12.45	9.66
	Vit C + PA	− 12.65	8.47	.669	− 37.17	11.85	− 15.57^*^	3.94	.002	− 26.97	− 4.17
	Placebo	− 21.99	8.30	.093	− 46.00	2.01	− 2.96	3.86	.972	− 14.13	8.19
	Placebo + PA^f^	− 5.99	8.57	.982	− 30.79	18.79	.68	3.98	1.00	− 10.84	12.22
Vit C^c^	Vit D + PA	21.11	8.52	.138	− 3.53	45.76	2.0	3.96	.994	− 9.46	13.46
	Vit C + PA	10.81	8.31	.784	− 13.23	34.86	− 14.18^*^	3.86	.005	− 25.36	− 2.99
	Placebo	1.481	8.13	1.00	− 22.05	25.01	− 1.57	3.78	.998	− 12.51	9.37
	Placebo + PA	17.47	8.41	.306	− 6.85	41.81	2.08	3.91	.995	− 9.23	13.40
Vit D + PA^b^	Vit C + PA	− 10.29	8.77	.848	− 35.65	15.05	− 16.18^*^	4.07	.002	− 27.97	− 4.38
	Placebo	− 19.63	8.60	.208	− 44.50	5.23	− 3.57	4.00	.941	− 15.13	7.99
	Placebo + PA	− 3.63	8.86	.998	− 29.26	21.99	.083	4.12	1.00	− 11.83	12.00
Vit C + PA^d^	Placebo	− 9.33	8.39	.876	− 33.61	14.93	12.60^*^	3.90	.012	1.31	23.89
	Placebo + PA	6.65	8.66	.972	− 18.39	31.71	16.26^*^	4.03	.001	4.61	27.91
Placebo^e^	Placebo + PA	15.99	8.49	.417	− 8.56	40.55	3.65	3.95	.931	− 7.76	15.07

Data are mean ± standard Error (SE)

PA, physical activity; BMI, body mass index; WC, waist circumferences; TC, total cholesterol; TG, triglycerides; LDL-C, low-density lipoprotein cholesterol; HDL-C, high-density lipoprotein cholesterol

* The mean difference is significant at the .05 level

^a^Receiving 2000 IU vitamin D per day

^b^Receiving 2000 IU vitamin D per day plus 30 min endurance physical activity

^c^Receiving 500 mg vitamin C per day

^d^Receiving 500 mg vitamin C per day plus 30 min endurance physical activity

^e^Receiving one placebo per day

^f^Receiving one placebo per day plus 30 min endurance physical activity

^g^Obtained from ANOVA, Tukey method

## Discussion

The results of this study show that the demographic characteristics of the participants do not differ significantly when comparing vitamin D to vitamin C treatment, suggesting that vitamin supplements may not have impact on demographic differences. However, elsewhere, studies indicate that vitamin supplements may have different functions in the body hence different age groups may have different needs for vitamin D supplement [38, 39]. The present study excluded participants who are likely having selected medical conditions hence agreeing with Gutierrez et al. [40] study who conducted vitamin D supplement intake among chronically ill patients and found that their vitamin supplement needs are different from healthy individuals. Our finding revealed that vitamin D and C supplements do not have any effect on weight when consumers are undergoing endurance physical exercise. The 12 weeks study did not report any difference in weight that is presumed vitamin D and C influence. Caan et al. [41] showed that vitamin D intake had a supplemental increase in postmenopausal weigh gain. Also, Zittermann et al. [42] showed that intake of vitamin D supplement enhances weight among cardiovascular risk patients. These two studies reported different results from the results of the present study. This may be due to the differences in the physical exercise variables used. In addition, previous studies focused more on supplemental intake of vitamin D as compared to the combination of vitamin C and D [43]. These studies also reported results from a specific population, which was excluded from the present study. Therefore, limited studies were found in the literature to evaluate the comparative effects of vitamin C and D supplement in weight loss and enduring physical exercise in MetS adult population in specific age group (30–50 years) as performed in this study. Furthermore, the current work found no significant interaction effect among participants taking vitamin D and C supplements with simultaneous exercise. However, interestingly, two statistical measures used in this study, one-way ANOVA and ANCOVA, showed different results. These differences were observed after baseline measure adjustment where ANCOVA demonstrated significant differences between BMI and the study vitamin supplement, similar finding was reported by Salehpour et al. [44], and Vimaleswaran et al. [45] reported a causal relationship between obesity and vitamin D, where they found a positive directional relationship between BMI and vitamin D. In our study, vitamin D may be responsible for the changes in BMI shown with ANCOVA analysis. This finding is in agreement with the result obtained by Amrein et al. [46].

Regarding, the changes in WC did not show any significant interaction among the study participants with vitamin D and C. The WC and BMI have been often studied together [47]. In line to our findings, a study performed by Du et al. [48] suggested that WC depends on high weight and individuals with low weight would not show any changes in their WC. Elsewhere, it has been reported that daily vitamin D supplementation (1000 IU) in overweight and obese women shows significant reduction in body fat mass with no effect on WC [44]. Overall, it appears that taking vitamin D alone do not have a significant effect on weight, but when it is combined with endurance PA, it may affect. However, further studies are required to fully observe this aspect. Furthermore, vitamin D supplement and its derivatives have widely been studied due to their connection with CVD [49]. Our results show that there is a significant difference in SBP among individuals who took vitamin D and did the exercise, while no significant interaction for vitamin C was recorded. This might due to the fact that vitamin D supplement aid in relieving blood pressure [50, 51]. However, study reported the audiometric outcomes of the use of vitamin D, suggested that the association of vitamin D and cardio related activities is uncertain; hence no clinical significance can be drawn [52]. In the present study, changes in the FPG did not have any significant interaction effects with vitamin D or C during exercise. Dakhale et al. [53] show that the FPG level for normal individuals should remain less than 250 mg/dL. Moreover, in this study, there was no significant effects on cholesterol levels among patients who took vitamin C supplements, but for vitamin D supplements cholesterol showed increased weight. The reason of vitamin D interaction with cholesterol in the MetS is high level functionality of vitamin D, which add bone mass, which interacts with adipose tissue or fats [50]. For the main study, TG had no significant interaction among study participants who took vitamin D and C supplements. The lack of interaction may due to the source of TG in the meals that participants were taking once a week to keep their TG low [54]. It was highly unlikely to report high level of TG among the study participants. Additionally, LDL-C and HDL-C had opposite results where LDL-C showed significant interaction with vitamin D compared to C, while HDL-C showed a significant interaction with vitamin C compared to D. The opposing results can be explained by differences in functional presence of either LDL-C or HDL-C within the body. Both vitamin C and D impact on metabolic functioning have been discussed in detail by Berge et al. [54], where vitamin bearing high responsibility for the additive and reductive changes in MetS. The main limitations of this study is its small sample size and duration is short which limits the generalizability of our results. However, studies with a larger sample size and longer follow-up period together with measurement of other related vitamins levels may yield more meaningful data on the effects of vitamins supplementations on MetS patients.

## Conclusions

We conclude that, consumption of vitamin D or vitamin C supplements may improves the life of metabolic syndrome patients. However, the combination of physical activities and vitamin supplements maximize the effect, and this combination should be recommended.

## Abbreviations

MetS: metabolic syndrome
CVD: cardiovascular disease
IDF: International Diabetes Federation
BMI: body mass index
PA: physical activity
RCT: randomized controlled trial
WC: waist circumference
FPG: fasting plasma glucose
TC: total cholesterol
TG: triglyceride
LDL-C: low-density lipoprotein cholesterol
HDL-C: high-density lipoprotein cholesterol
